# Morphosyntactic Skills Influence the Written Decoding Accuracy of Italian Children With and Without Developmental Dyslexia

**DOI:** 10.3389/fpsyg.2022.841638

**Published:** 2022-04-27

**Authors:** Emanuele Casani, Mila Vulchanova, Anna Cardinaletti

**Affiliations:** ^1^Department of Linguistics and Comparative Cultural Studies, Università Ca’ Foscari Venezia, Venice, Italy; ^2^Department of Language and Literature, Faculty of Humanities, Norwegian University of Science and Technology, Trondheim, Norway

**Keywords:** reading, developmental dyslexia, dual-route model, dual-route approach to orthographic processing, morphology, syntax, clitic pronouns

## Abstract

Some types of developmental dyslexia (DD) are associated with morphology. Deep DD leads to morphological and semantic errors, and possible comorbidity with syntactic deficits; phonological-output-buffer DD causes problems in decoding longer morphologically complex words. In addition, cross-linguistic studies highlight the effects of morphological awareness on reading accuracy and fluency. The role of morphosyntactic abilities on reading is, however, not clear. This study explores the influence of morphosyntactic competence on reading in Italian children with and without DD. A total of 14 children with DD and 28 with Typical Development (TD) attending the Italian primary school were tested on written decoding, syntactic comprehension of different grammatical structures, and syntactic production of direct object clitic pronouns. DD children were significantly less accurate and slower in reading than TD children. Syntactic skills of the two groups did not differ significantly, but some differences in their acquisitional pace emerged. Syntactic comprehension and production of direct-object-clitic pronouns predicted reading accuracy standard scores, thus suggesting that morphosyntactic abilities, beyond clitics’ weak phonological status, affect decoding accuracy. Decoding accuracy was influenced by reading errors related to morphology (morphological, semantic, and phonological-output-buffer errors). Decoding speed was a specific weakness of DD children and was rather affected by multi-letter combinations. Consistent with a *dual-route approach to orthographic processing*, we argue that accuracy depends on *fine-grained* decoding strategies maximizing the precise ordering of letters, thus it is more sensitive to morphosyntactic skills. Morphological reading errors were associated with phonologically weak (determiners, clitic pronouns, and prepositions) and salient words (verbs). This suggests that the decoding of function words and morphologically complex words is particularly demanding and related to both phonological and morphosyntactic skills. Age had a negative predictive effect on semantic errors, compatible with the gradual acquisition of lexical decoding strategies, which seemed to be slowed down by DD. We conclude that oral morphosyntactic skills play a role in reading accuracy in the Italian shallow orthography for both DD and TD children. It is then advisable to assess children’s linguistic profile during DD diagnoses to establish whether some reading errors are related to morphosyntactic weakness. In this case, *ad hoc* morphosyntactic training might support reading accuracy.

## Introduction

Reading is a complex activity that involves several underlying abilities including language, metalanguage, and cognitive skills (cf. Nagy and Townsend, 2012). In this study, we bring evidence of the role of morphosyntactic skills on decoding accuracy in Italian children with and without developmental dyslexia (henceforth, DD). We also show that some decoding errors that have to do with morphology to different degrees play a central role in decoding accuracy.

### Dual-Route Model of Reading

**FIGURE 1:**
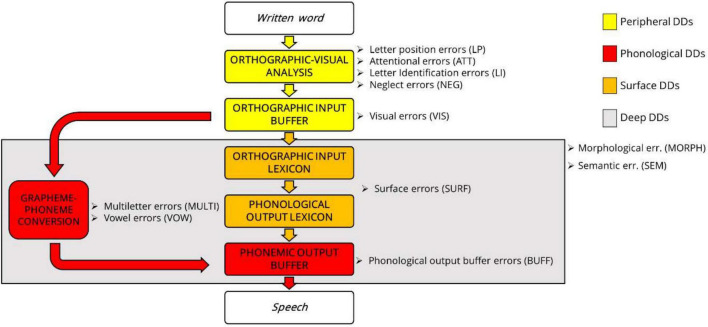
Dual-route model of reading aloud, with different error types associated with each section of the model, and consequential types of DD. Yellow cases display the visual-orthographic step of reading; orange cases display the lexical route; and red cases display the sublexical route of reading, including the phonological output buffer (Casani, 2021, adapted after Friedmann and Coltheart, 2018).

There is wide consensus on the fact that an accurate model to describe typical reading aloud processes should include a lexical and a sublexical route (Coltheart et al., 2001; for a review, see Castles, 2006; for a case study arguing for the existence of a third route of reading, see Wu et al., 2002). The lexical route allows reading by accessing the lexicon for previously seen written words stored in long-term memory, whereas the sublexical route uses a set of mapping rules to convert graphemes into phonemes, thus allowing the reading of every regular word, both known and unknown, in particular in shallow orthographies. At the top of the dual-route model, there is a common stage of orthographic visual analysis, which is responsible for letter identification, encoding of letter position within the word, and binding of letters to words. In the last stage, the phonological string generated through either the lexical or sublexical route is sent to the phonological output buffer, a short-term interface between phonological representations and articulatory motor programming having the function to keep the information until full production and to assemble phonological strings into larger units (Zoccolotti et al., 2005; Castles, 2006; Friedmann and Coltheart, 2018). Recent studies reveal that different decoding error types can be associated with atypical functioning or non-functioning of specific sections of the dual-route model of reading, which can give rise to different types of DD (Friedmann and Coltheart, 2018), even in a shallow-orthography language like Italian (Traficante et al., 2017). Figure 1 displays the dual-route model of reading, decoding errors associated with the different sections of the model, and related types of DD.

Some decoding error types have to do with morphology to different extents. In particular, morphological and semantic decoding errors can be associated with a deficit in both the lexical and sublexical route of reading, resulting in deep DD (Stuart and Howard, 1995). In this condition, words that can be read via meaning by resorting to their visual imagery properties are generally preserved, whereas function words and morphologically complex words are particularly problematic. The difficulty with function words might depend on their abstractness, which makes them particularly difficult to be imagined (Friedmann and Coltheart, 2018). Function words can be replaced with visually similar lexical words, other function words, or just omitted. The *imageability effect* could also determine the difficulty with morphologically complex words, which can be decomposed into lexical (bases, stems, or roots) and functional chunks (morphological affixes) requiring the co-activation of different sections of the reading model. In particular, bases, stems, or roots might be read by resorting to the semantic lexicon, whereas affixes via a direct (lexical) route linking the orthographic input buffer to the phonological output buffer (see Figure 1). If this route is impaired, morphologically complex words can be simplified through omissions and substitutions of morphological affixes (Friedmann and Coltheart, 2018). Children with deep DD can also present with syntactic deficits, which might make it difficult to resort to the context in reading (Friedmann and Coltheart, 2018).

A deficit in the phonological output buffer (phonological-output-buffer dyslexia) can be responsible for errors in decoding longer morphologically complex words, characterized by omissions, substitutions, and transpositions of some phonemes.

The possible presence of syntactic deficits in children and adults with DD (Bishop, 1991; Muter and Snowling, 1998; Talli et al., 2013; Cardinaletti and Volpato, 2015; Friedmann and Coltheart, 2018) suggests that these deficits might contribute to difficulties in retrieving function words and morphological affixes, which convey morphosyntactic information, during reading. In particular, we wonder whether the three error categories described above (morphological, semantic, and phonological-output-buffer errors) might be influenced by the reader’s morphosyntactic skills. Namely, whether adequate morphosyntactic competence might improve children’s familiarity with text chunks encoding morphosyntactic information, thus allowing faster retrieval through the activation of the direct (lexical) route from the orthographic input lexicon to the phonological output buffer. At the same time, good morphological knowledge might help individuals with DD in accessing subparts of morphologically complex words, thus benefitting from the cumulated frequency of morphemes to process shorter inputs by co-activation of the direct lexical route beside the semantic lexicon.

### A Dual-Route Approach to Orthographic Processing

By applying the dual-route model of reading to a smaller scale of granularity, Grainger and Ziegler (2011) propose a dual-route approach to orthographic processing. They suggest that optimization of print-to-meaning mapping takes place thanks to two distinct learning constraints based, respectively, on the prelexical orthographic coding processes of *diagnosticity* and *chunking*.

*Chunking* allows the detection of relevant letter combinations corresponding to pre-existing sublexical phonological and morphological representations. It takes place along the *fined-grained* route, which is activated by frequently co-occurring contiguous letter combinations with a precise letter ordering, and precise placement with respect to the beginning and ending of words. These include prefixes and suffixes, which are subject to morpho-orthographic processing.

On the other hand, *diagnosticity* allows for the selection of letter combinations that are informative with respect to word identity. It takes place along the *coarse-grained* route, which codes for approximate letter position within words, irrespective of letter contiguity. This route benefits from the combinations of most visible letters that best constrain word identity. So, it provides a lower precision level in coding letter-position information compared to the fine-grained route, but a higher speed level because it can provide faster top-down activation of whole-word representations. In skilled readers, when most visible letters combined with contextual constraints are not sufficient to activate top-down constraints, the fine-grained route intervenes to disambiguate the information.

Parallel development and smooth integration of the two routes enable the emergence of morpho-semantic and morpho-orthographic representations as well as increased sensitivity to morphological structure, thus reducing the effects of word length and phonological recoding (Grainger and Ziegler, 2011). Since the detection of morphological constituents is the key mechanism of morpho-orthographic chunking (which is performed along the fine-grained route), we suppose that higher morphological competence should facilitate the detection of morphological constituents, thus increasing the reading performance, in particular concerning accuracy. In the following section, we report evidence that morphology influences word decoding through automatic morpho-orthographic segmentation.

### Morphological Awareness and Reading

Besides the vast literature around phonological awareness, orthographic competence, and rapid automatized naming (RAN) (for a review, see Casani, in preparation, 2021), cross-linguistic research provides evidence of the role of morphological awareness as a predictor of word reading accuracy (Burani et al., 2008; Traficante et al., 2011), fluency (Fowler and Liberman, 1995; Carlisle and Katz, 2006; Roman et al., 2009), and comprehension (Deacon and Kirby, 2004; Nagy et al., 2006; Tong et al., 2011). Verhoeven and Perfetti (2011) highlight the role of morphology in reading across a wide range of languages, and suggest that morphology, “which is foundational for language knowledge, is universally part of reading, subject to constraints imposed by the language and by how the writing system encodes that language.” Besides the universal phonological principle that all writing systems support the activation of phonology at their smallest functional grapheme units (e.g., Perfetti, 2003), they suggest that cross-linguistic research might lead to a universal morphology principle. The ease of word identification and the role of morphology may vary across languages depending on their orthographic depth (Frost et al., 1987) and their morphological richness (Vannest et al., 2002).

According to the *orthographic depth hypothesis* (Frost, 2006), the opaque relationship between phonemes and graphemes in deep orthographies is handled by resorting to lexical mediation. The extent of involvement of lexical mediation is determined by the orthographic depth of the language. Before learning to read, words are stored as holistic phonological units. As literacy is acquired, these bigger phonological representations gradually give way to syllable and then phoneme representations and determine a restructuring of the learner’s lexicon granularity. According to the *grain size theory* (Frost et al., 1987), phonology offers a bigger scale and orthography a smaller scale of granularity, which are represented by phonological units and letters, respectively. The degree of consistency between phonemes and letters might determine the speed of reading development (Vulchanova and Farukh, 2018).

In Italian, a shallow orthography with high grapheme-phoneme consistency, morphological information has been shown to influence both reading fluency (Burani, 2010) and accuracy (Angelelli et al., 2014). Children of different reading ages take advantage of morphemic lexical units (Burani et al., 2002). “Morphemes may develop as orthographic and phonological salient reading units” (Burani et al., 2008, p. 254), and these “common letter patterns might become consolidated in lexical memory” (Deacon et al., 2011, p. 476). Masked-priming experiments (e.g., Meunier and Longtin, 2007) confirm that skilled adult readers possess orthographic representations that are structured morphologically and activated before representations of whole words. How this (quasi-)regular trend influences the acquisition of reading across languages is not clear.

There is cross-linguistic evidence (Quémart et al., 2011; Beyersmann et al., 2012; Dawson et al., 2018) that adult skilled readers process complex words and non-words based on morphological structure. Masked priming experiments across English (Beyersmann et al., 2012) and French (Beyersmann et al., 2015) showed robust morphological priming effects on word recognition for child participants, but only when morphological primes had a semantically transparent relationship with targets (e.g., darkness-DARK). Beyersmann et al. (2012) found no evidence that English children aged 8–10 use morpho-orthographic analysis: priming effects for pseudo-morphological pairs (e.g., corner-CORN) could not be distinguished from those based on non-morphological form overlap (e.g., brothel-BROTH). In a related comparison, Beyersmann et al. (2015) could not differentiate masked priming effects for suffixed non-word pairs (e.g., tristerie-TRISTE) and non-suffixed non-word pairs (e.g., tristald-TRISTE) in French readers aged 7–11 (see also Hasenäcker et al., 2016, for a similar study in German). The developmental trajectory observed in English (Beyersmann et al., 2012) also appears in Hebrew, a Semitic language with a very rich morphological structure (Schiff et al., 2012).

In Italian, morphological awareness can affect decoding since the second grade (Burani et al., 2002, 2008; Marcolini et al., 2011; Traficante et al., 2011). Interestingly, a facilitating effect on word reading speed was observed in second-graders and children with DD, whereas in older skilled readers it was limited to low-frequency words. Furthermore, a cross-linguistic study on English and French (Casalis et al., 2015) suggests a higher degree of morphological processing efficiency in French (affecting both accuracy and latencies in a lexical decision task) than in English (affecting accuracy only).

Studies on morphologically productive languages like French (Quémart et al., 2011) and Hebrew (Schiff et al., 2012) provide evidence of morpho-orthographic decomposition in young readers as in adults, differently from English children aged 7–10 years (Beyersmann et al., 2012). Burani et al. (2002) report that Italian children aged 8–10 read aloud morphologically structured non-words more quickly and accurately than non-words without morphological structure. In a similar reading aloud experiment, children aged 9–11 read aloud morphologically complex English words with a high-frequency stem more quickly and accurately than those with a lower-frequency stem (Deacon et al., 2011). In a lexical decision task, Casalis et al. (2015) report that English and French children between the ages of 7 and 10 found morphologically structured non-words (e.g., *gifter*) harder to reject than non-words without a morphological structure (e.g., *curlip*). The same pattern was reported by Burani et al. (2002) for Italian children of similar age, thus replicating the pattern observed in skilled readers (Crepaldi et al., 2010).

In more recent research using this paradigm with three age-groups of developing English readers (ages 7–9, 12–13, and 16–17), the two younger groups showed an effect of morphological structure on accuracy, whereas only older adolescents (16–17 years old) and adults showed this effect on reaction time (Dawson et al., 2018).

Häikiö et al. (2011) examined the role of morphology in Finnish reading development by measuring participants’ eye movements while they read sentences containing either a hyphenated (e.g., *ulko-ovi* “front door”) or concatenated (e.g., *autopeli* “racing game”) compound. The participants were Finnish second, fourth, and sixth graders. Fast second graders and all fourth and sixth graders read concatenated compounds faster than hyphenated compounds. This suggests that they resort to slower morpheme-based processing for hyphenated compounds but prefer to process concatenated compounds via whole-word representations.

Further eye-tracking research showed that eye movements are affected by both whole-word frequency and first-constituent frequency. In processing Dutch (Kuperman et al., 2008, 2009) and Italian (Marelli and Luzzatti, 2012) compounds, frequency in first-fixation duration, namely the time initially spent by the reader fixating the target element, correlates negatively with the frequency of whole-words.

In processing Italian derived words, stem frequency has a facilitating effect on first-fixation duration only within sentences prompting a semantically transparent interpretation of the word, whereas a stem-frequency effect is inhibitory within sentences prompting an opaque interpretation of the target word (Amenta et al., 2015). Word frequency, as well as the amount of information and the size of the morphological family of the suffix, affects the reading times of Dutch derived words with shorter suffixes. This is interpreted as a *relative entropy* effect of morphemes. Affixes occurring more frequently are more salient and processed faster (Kuperman et al., 2010).

In English, root frequency affected fixation times for longer (about eight letters) but not shorter (about six letters) prefixed words, whereas whole-word frequency for shorter but no longer prefixed words (Niswander-Klement and Pollatsek, 2006). In Italian, base and word frequency affected first-fixation duration for nouns derived from noun bases differently: base frequency facilitated first fixation, whereas word frequency had an inhibitory effect (Traficante et al., 2018).

Behavioral data from languages with rich morphology show differences in lexical decision times for nouns, adjectives, and verbs. Kostić and Katz (1987) attribute this effect in Serbo-Croatian to the number of inflectional alternatives available for each grammatical class. Deutsch et al. (1998) ascribe the differences in processing verbs and nouns in Hebrew, beyond semantic and syntactic components, to the distributional properties of constituents, namely to the fact that “when a morpheme is common to more words in the language, its impact on processes of morphological decomposition is prominent” (p. 1,252). Italian skilled adult readers recognized verbs slower than nouns and adjectives. Moreover, latencies for verbs, but not for nouns or adjectives, correlated with their base frequency (Colombo and Burani, 2002; Traficante and Burani, 2003).

Marcolini et al. (2011) showed that Italian children with DD read pseudowords made up of a root and a derivational suffix faster and more accurately than simple pseudowords. However, only dyslexic and reading-matched younger children benefited from morphological structure in reading words aloud. The authors investigated the effects of word frequency and word length on complex-word reading in Italian dyslexic and skilled readers and showed that word frequency affects the probability of morpheme-based reading, interacting with reading ability. Young skilled readers named polymorphemic words faster than simple words only when they were of low frequency, whereas they read high-frequency polymorphemic words as fast as high-frequency simple words. By contrast, poor readers took advantage of polymorphemic words irrespective of word frequency, while adult readers showed no facilitating effect of morphological structure. Similar findings emerged in English (Carlisle and Stone, 2005) and Danish (Elbro and Arnbak, 1996) populations, where only younger and dyslexic children read derived words faster than monomorphemic words, whereas morphological complexity did not affect reading speed in the elder skilled children. This indicates that morpheme-based reading is effective for both poor and skilled young readers when a whole-word representation is not firmly established in the reader’s orthographic lexicon, because either the whole word is not familiar to the reader or (s)he has poor reading skills (Marcolini et al., 2011).

Angelelli et al. (2014) found that morphological information in Italian is a useful resource for both reading and spelling, as typically developing children benefit from the presence of morphological structure when they read and spell non-words. In processing low-frequency words, however, morphology facilitates reading, but not spelling. They attribute their results to successful cooperation between lexical and sublexical processes in reading and spelling, which facilitate morpho-lexical access.

These data converge on the fact that morphological awareness facilitates lexical reading for low-frequency words that otherwise would not probably be represented as a whole in the mental lexicon, even in Italian and other shallow orthographies (for Spanish, see Defior et al., 2008; Suárez-Coalla et al., 2017), where the orthography-phonology mapping might be expected as sufficient for correct decoding and spelling. This suggests that morphological competence and its interface with syntactic competence play a role in written decoding.

### (Morpho)syntactic Competence and Reading

Syntactic competence has been generally explored in relation to reading comprehension, of which it is deemed as a good predictor (see for instance, Simpson et al., 2020 for Spanish speakers; Morvay, 2012 for speakers of English as a foreign language; Chik et al., 2012 for Chinese speakers). Fewer studies analyzed its effects on decoding. Sana Teixeira et al. (2016) revealed relations of syntactic awareness with both reading accuracy and reading speed in typically developing Brasilian children in primary school (age: 9;0–11;7).

Traficante et al. (2018) analyzed the role of the base word distributional properties on eye-movement behavior and found an inhibitory base frequency effect, but no word frequency effect for nouns derived from verb bases. They suggest that syntactic context, calling for a noun in the target position, is responsible for the inhibitory effect when a verb base is detected, thus hampering the lexical access to the corresponding base-suffix combination.

A recent longitudinal study (Casani, in preparation, 2021), besides confirming a strong predictive role of oral syntactic comprehension on reading comprehension, found that syntactic comprehension, measured in the last year of kindergarten and second grade of primary school through the Italian version of Bishop (2009), predicted both word and text decoding accuracy in second grade. In particular, it was a predictor of surface errors, which are related to the lexical route of reading. This highlights the strict relation between (morpho)syntactic and lexical competence, as confirmed by the correlations of syntactic comprehension with both receptive (ϱ = 0.540, *p* = 0.000) and productive (ϱ = 0.554, *p* = 0.000) vocabulary. In the same research, longitudinal syntactic-comprehension skills predicted the emergence of difficulties in word and non-word writing, beyond reading comprehension difficulties, in second and third grade; longitudinal syntactic production of third-person direct object clitic pronouns predicted the emergence of decoding accuracy and decoding speed difficulties.

These data find support in the study of event-related potentials in adult speakers of German (Cantiani et al., 2013), a morphologically rich language with relatively shallow orthography like Italian. Seventeen subjects with DD and seventeen with TD were presented with oral stimuli with morphosyntactic violations. DD participants showed anomalous morphosyntactic processing, especially when morphosyntactic violations were expressed by both lexical and inflectional changes. Furthermore, anomalous morphosyntactic processing was mediated by lexical cues instead of acoustic salience. Several behavioral studies also report the presence of syntactic deficits in subjects with DD (Bishop, 1991; Muter and Snowling, 1998; Talli et al., 2013; Cardinaletti and Volpato, 2015).

### The Current Study

The literature mentioned in previous sections shows that some decoding errors related to the central routes of reading (i.e., the lexical and sublexical route) have to do with morphology to different extents. Moreover, morphological awareness and its interface with syntax have a prominent cross-linguistic role in reading accuracy and speed. Yet, only few studies have investigated the influence of general (morpho)syntactic competence on written decoding.

In the present study, we analyze the effects of oral (morpho)syntactic comprehension and production, as well as different reading errors, including morphological ones, on the written decoding of Italian primary-school children with and without DD.

We expected to find effects of (morpho)syntactic competence on decoding accuracy, due to greater familiarity with morpho-lexical chunks and distributional properties, by children with higher morphosyntactic skills. We did not expect the same effects on decoding speed. In fact, according to the dual-route approach to orthographic processing (Grainger and Ziegler, 2011), speeding up reading processes in skilled readers depends on the ability to process not only *fine-grained* orthographic strings preserving the information about letter ordering, as morphemes are, but also *coarse-grained* orthographic representations, which code for the presence of informative letter combinations in the absence of precise positional information (Traficante et al., 2018).

## Materials and Methods

### Participants

A total of 53 Italian monolingual children in primary school were initially tested (for the results of the whole sample, see Casani, 2020a,b). They were recruited in primary schools in the Center and South of Italy. In total, 11 of them were excluded due to the presence of language disorders or the alleged presence of developmental problems based on teachers’ reports. Among the 42 participants (24 females + 18 males), one child was in second grade, 16 children were in third grade, 8 were in fourth grade, and 17 were in fifth grade.

A total of 14 children [age 7;5–10;9 (*M* = 9;9, *SD* = 0;11)] had a diagnosis of general DD, and 28 [age 8;4–11;3 (*M* = 9;6; *SD* = 0;11)] were age-matched Typically Developing (TD) children. Diagnoses were established by the Italian public health system (ASL) or authorized private clinical centers. Children included in the TD group were not reported by teachers for any language or learning problems.

### Materials and Procedures

The participants’ families signed informed parental consent. Children expressed their willingness to participate in the activities during an exploratory interview. The procedures followed the ethical principles of the Declaration of Helsinki. Children were tested individually in silent and adequately lit rooms in school facilities. Tests were administered by the first author. Different abilities including syntactic comprehension, syntactic production, and reading were tested. Syntactic comprehension was tested through a standardized picture-sentence matching task extracted from the BVN 5–11 (Neuropsychological Assessment Battery for the developmental age) (Bisiacchi et al., 2005). It is a reduced adaptation of Bishop (2009) consisting of 18 items investigating the comprehension of different syntactic structures (for additional information, see Supplementary Data).

Syntactic production was tested through a non-standardized elicitation task of third-person direct object clitic pronouns (Arosio et al., 2014). These are complex structures requiring the mastering of phonological, morphosyntactic, syntactic, and pragmatic skills. The test elicits 12 third-person singular direct object clitic pronouns (6 masculine + 6 feminine) under conditions of gender and number match with the sentential subject. Casani and Cardinaletti (2021) recently showed that this morphosyntactic combination is significantly more accessible than combinations including gender mismatch between the clitic pronoun and the sentential subject. Children were shown two-slide cartoons, where the recorded voice of an Italian male native speaker presented the situation through a brief sentence [e.g., *In questa storia c’è un signore che vuole pescare un pesce* (In this story, there is a man who wants to fish a fish)] and then asked a question [*Guarda! Cosa sta facendo al pesce?* (Look! What is Ø doing to the fish? → Look! What is he doing to the fish?)]. The restrictive context should elicit a null-subject sentence containing a third-person direct object clitic pronoun agreeing in gender and number with its antecedent (*Lo__3r*d.sing.masc.dir.clit*_. sta pescando.* (Ø it*__3r*d.sing.masc.dir.clit*_* is fishing. → He is fishing it). Grammatical and pragmatically appropriate responses were assessed as correct. The test was administered through a 15-inch laptop screen with stereo speakers.

Decoding accuracy and speed were tested through standardized texts calibrated to the students’ grades. These stem from the MT-2 battery (Cornoldi and Colpo, 2011). They were black printed on A4 white paper.

### Analyses

#### Syntactic Comprehension

To our aims, we opted to only analyze the effects of grammar-focused items. According to the authors of the test, items 1–8 are focused on lexicon, whereas items 9–18 are focused on grammar. We do not agree with considering item number 5 (*La mucca le sta guardando* [“The cow them is watching” = The cow is watching them)] as a lexicon-focused item because it includes the interpretation of a third-person direct object clitic pronoun, a structure requiring high-level morphosyntactic and syntactic skills (for the difficulties involved in third-person direct object clitic pronouns, see Arosio et al., 2014; Casani, 2020c). We then included it among grammar-focused items and analyzed it as such. The complete list of the structures analyzed is in Supplementary Data. Correct responses (pointing to the right picture) were assigned one point; incorrect responses were assigned 0 points. Proportional scores were analyzed.

#### Syntactic Production

Responses containing a grammatical third-person direct object clitic pronoun were assigned one point. Ungrammatical responses (gender or number errors in clitic agreement, clitic omissions, clitic-position errors) and sentences containing a full determiner phrase instead of the clitic (which is grammatical but pragmatically inappropriate) were assigned 0. Proportional scores were analyzed.

#### Reading

The total error number and the speed rate (syllables per second) were computed and converted into standard scores. Accuracy and speed standard measures (*Z*) were analyzed.

An analysis of proportional reading errors based on an adaptation of the coding scheme by Friedmann and Coltheart (2018) was performed. Eleven types of decoding errors were detected, as shown below.

1. LP (Letter Position errors), e.g., dispiacere → *despicare; presso → perso.
2. ATT (ATTentional errors), e.g., dal tetto → dal letto; se mi hai letto → se mai hai letto.
3. LI (Letter Identity errors), e.g., due → bue; babbo → *papo.
4. NEGL (NEGLect errors), e.g., rallegrò → allegro; bigi → *bi.
5. VIS (VISual errors), e.g., nipotino → *nipotivino; fradicia → *fraggida.
6. SURF (SURFace errors), e.g., [’]*fradicia*–*fra[’]*dicia*; *si* [’]*presero* → si *pre[’]*sero.*
7. MULTI (MULTIletter errors), e.g., *cresceva*–*crescheva; *foglio* →*forgerio.
8. VOW (VOWel errors), e.g., dimissioni–*dimessioni; a lungo → e lungo.
9. MORPH (MORPHological errors), e.g., *dimenticando* → *dimenticato*; *sentì* → *sente.*
10. SEM (SEMantic errors), e.g., *ripeté* → *ribatté*; *a bocca aperta* → *a mano aperta.*
11. BUFF (phonological-output-BUFFer errors), e.g., *ringraziamenti* →*rangrizzamenti; *sentenziava* → *sensiva.

Error categories 1, 2, 3, 4, and 5 are related to the orthographic-visual-analysis stage of reading, at the top of the reading model; category 6 is related to the lexical route of reading; categories 7 and 8 are related to the sublexical route of reading; categories 9 and 10 are related to both central routes, namely the lexical and sublexical routes; category 11 is related to the phonological output buffer, at the bottom of the reading model (see Figure 1).

Analyses of the elements involved in different error types (adjectives, adverbs, clitic pronouns, conjunctions, determiners, nouns, prepositions, pronouns, verbs, whole phrases) and of superficial errors (additions, omissions, changes, moves, and substitutions with a different part of speech) associated with different error types were performed.

### Statistical Analyses

Two generalized mixed models (GMM) were run to analyze the effects of group, syntactic comprehension, and syntactic production on standard measures of decoding accuracy and speed. Decoding errors were entered as random effects. This allowed us to control simultaneously for the effects of all error types.

In addition, we ran 11 GMMs to analyze the effects of the same factors/variables on each error type.

As the distribution of children across grades was not homogeneous (for the number of participants in each grade, see Supplementary Tables 1, 4), we opted to enter age in months as a random effect, which allowed us to control for a more analytical measure than the grade variable.

In addition, we ran two GMMs to analyze the combined effect of age and group (age variable nested in the group variable) on syntactic comprehension and production, respectively; and two GMMs, where we replaced the age variable with grade (nested in the group variable).

We finally checked the combined effect of grade and group (grade variable nested in the group variable as a fixed effect) on SEM-error proportions (dependent variable).

Fisher’s exact tests with *post hoc Z* (*Bonferroni*) were used to analyze the association between each error type and different parts of speech.

Statistical analyses were run in SPSS-24 and are described in detail in section “Results.”

## Results

### Differences Between Groups

**FIGURE 2:**
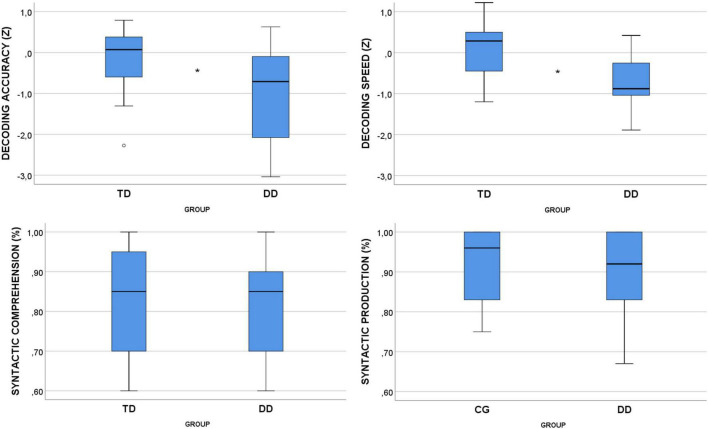
Distribution of decoding standard scores and syntactic percent scores (*p < 0.05).

Figure 2 displays the distribution of standard scores obtained in decoding, and of percentage scores obtained in syntactic tests by TD and DD children.

Four distinct GMMs with scores obtained on syntactic (comprehension and production) and decoding (accuracy and speed) tests as respective dependent variables, the double level of group as a fixed effect, and children’s age (in months) as a random effect revealed a significant effect of group on decoding accuracy [*F*(1, 40) = 8.584, *p* = 0.006] and speed [*F*(1, 40) = 16.727, *p* = 0.000]. TD children were significantly more accurate and faster than DD-children, as shown in Table 1.

**TABLE 1:** Differences between TD and DD children in standard measures of decoding accuracy and speed.

					*CI* (95%)	
Outcome	*Coeff*. (TD)	*SE*	*t*	*p*	Lower	Upper
Decoding accuracy (*Z*)	0.820	0.280	2.930	0.006	0.254	1.385
Decoding speed (*Z*)	0.868	0.212	4.090	0.000	0.439	1.297

No significant effect of group emerged on syntactic comprehension (*p* = 0.399) and syntactic production (*p* = 0.535). The significance of the random effect was also analyzed and revealed no effect of age on any variable (0.259 < *p* < 0.697).

#### Effects of Grade on Morphosyntactic Skills

As we analyzed (morpho)syntactic proportional scores instead of standard scores,^1^ we verified in more depth the absence of effects of age on morphosyntactic skills by running two robust GMMs with syntactic comprehension and production scores as respective dependent variables, and the age variable nested in the group variable as a fixed effect. Age (combined with group) confirmed no predictive effect on syntactic comprehension (*p* = 0.184) and production (*p* = 0.187).

Then, we ran two additional models by replacing the age variable with grade. Grade (nested in the group variable) predicted both syntactic comprehension [*F*(6, 33) = 95.269, *p* = 0.000] and production [*F*(6, 31) = 14.446, *p* = 0.000].

As for morphosyntactic comprehension (for complete statistics, see Supplementary Tables 1–3), there was a significant score increase in the TD group between third and fourth grade (*Est* = 0.182, *SE* = 0.043, *p* = 0.000).^2^ In fourth grade only, the TD-group’s score was significantly higher than that of the DD group (*Est* = 0.168, *SE* = 0.069, *p* = 0.020).

As for morphosyntactic production (for complete statistics, see Supplementary Tables 4, 5), there was a significant increase in target clitic pronouns between fourth and fifth grade in the DD group only (*Est* = 0.154, *SE* = 0.065, *p* = 0.024). No differences between groups emerged.

### Predictors of Reading

Two GMMs were run with decoding accuracy and decoding speed as the respective dependent variables, the double level of group (TD and DD), syntactic-comprehension and syntactic-production scores as fixed effects, and age (in months) and the 11 decoding error types as random effects. The models significantly predicted decoding accuracy [*F*(3, 38) = 8.477, *p* = 0.000] and decoding speed [*F*(3, 38) = 4.297, *p* = 0.010].

Significant main effects of group [*F*(1, 38) = 4.494, *p* = 0.041], syntactic comprehension [*F*(1, 38) = 14.137, *p* = 0.001], and syntactic production [*F*(1, 38) = 6.716, *p* = 0.013] emerged on decoding accuracy, whereas only a main effect of group emerged on decoding speed [*F*(1, 38) = 12.247, *p* = 0.001].

Significant fixed effects are reported in Table 2.

**TABLE 2:** Predictive fixed effects of group and syntactic skills on decoding standard scores.

						*CI* (95%)	
Dependent variable	Predictor	*Coeff.*	*SE*	*t*	*p*	Lower	Upper
Decoding accuracy	Group (TD)	0.284	0.134	2.120	0.041	0.013	0.556
	Syntactic comprehension	1.684	0.448	3.760	0.001	0.777	2.591
	Syntactic production	0.591	0.228	2.591	0.013	0.129	1.052
Decoding speed	Group (TD)	0.687	0.196	3.500	0.001	0.290	1.085

TD children [*M* = 0.604, *SE* = 0.745, *CI* (−0.905, 2.113)] were significantly more accurate than DD-children [*M* = 0.320, *SE* = 0.754, *CI* (−1.206, 1.845)]; TD children [*M* = 0.314, *SE* = 0.408, *CI* (−0.512, 1.139)] were also faster than DD-children [*M* = − 0.374, *SE* = 0.432, *CI* (−1.248, 0.501)], after controlling for age and decoding errors.

Analyses of random effects revealed that BUFF, MORPH, and SEM errors affect decoding accuracy, whereas MULTI errors affect decoding speed significantly. Significant random effects of decoding errors are reported in Table 3.

**TABLE 3:** Significant random effects of percent decoding errors on standard measures of decoding accuracy and speed.

						*CI* (95%)	
Dependent variable	Effects	*Est.*	*SE*	*df*	*p*	Lower	Upper
Decoding accuracy	BUFF	–0.987	0.337	38	0.006	–1.670	–0.304
	MORPH	–10.929	1.502	38	0.000	–13.970	–7.889
	SEM	–6.259	1.047	38	0.000	–8.379	–4.138
Decoding speed	MULTI	–4.756	1.872	38	0.015	–8.546	–0.966

#### Morphosyntactic Predictors of Reading Accuracy

A GMM with decoding accuracy as the dependent variable, the 11 syntactic-comprehension items focused on grammar (including item number 5, which was erroneously coded as a lexicon-focused item by the test authors, as explained in section “Materials and Methods”) as fixed effects, and group and age as random effects was run with a stepwise procedure. The model correctly predicted decoding accuracy standard scores [*F*(4, 13) = 3.258, *p* = 0.047]. Item number 5, namely a sentence requiring the interpretation of a third-person feminine plural direct object clitic pronoun (see section “Materials and Methods”), was a significant predictor of decoding accuracy [*Coeff*. = 1.125, *SE* = 0.438, *t* = 2.566, *p* = 0.023, *CI* (0.178, 2.072)].

### Reading Errors

**FIGURE 3:**
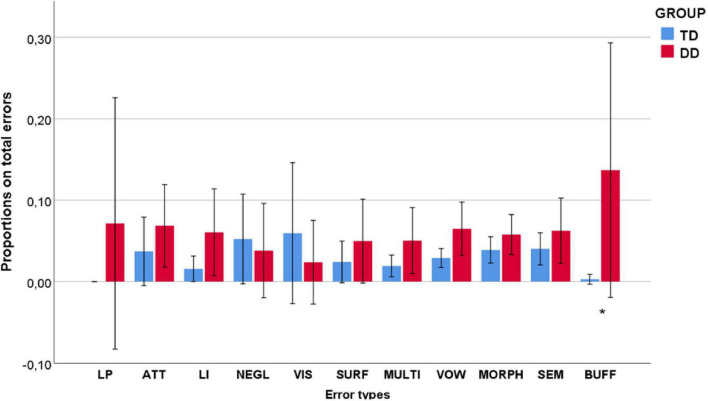
Proportions of decoding errors by TD and DD children (*CI* = 95%) (*p < 0.05).

Figure 3 displays the percentages of reading errors made by TD and DD children.

LP-errors (7%) were present only in DD-children. Errors were numerically higher in DD-children than in TD children for every category except VIS (TD = 6%; DD = 2%) and NEGL errors (TD = 5%; DD = 4%). These two error types showed high *SD* (VIS = 0.189; NEGL = 0.129). Fisher’s exact test revealed a significant association between individuals and error types (*Fisher* = 437.235, *V* = 0.393, *p* = 0.000). *Post hoc* analyses revealed that significant rates of VIS (0.70%; *Z* = 4.0) and NEGL (1.10%; *Z* = 4.0) errors were associated with two different children of the TD group (Bonferroni, *p* ≤ 0.050).

A series of 11 robust GMMs with each decoding error type as a dependent variable, group as a fixed effect, and age (in months) as a random effect correctly predicted BUFF errors [*F*(1, 40) = 10.104, *p* = 0.003]. TD children made significantly fewer BUFF errors than DD children [*Coeff*. = −0.137, *SE* = 0.043, *t* (40) = −3.179, *p* = 0.003, *CI* (−0.224, −0.050)].

#### Morphological and Semantic Errors

Fisher’s exact test revealed a significant association between error categories and parts of speech (*V* = 0.303, *p* = 0.000). *Post hoc* analyses revealed that MORPH decoding errors are significantly associated with determiners (87.5%, *Z* = 4.6), clitic pronouns (67.9%, *Z* = 3.9), prepositions (63.6%, *Z* = 3.0), and verbs (43%, *Z* = 2.1). Significant rates of MORPH decoding errors (*Fisher* = 165.505, *V* = 0.451, *p* = 0.000) consisted of substitutions with other visually similar parts of speech [e.g., *le mostrò* → *e mostrò*; *ebbe finito* → *ebbe fino; *in compenso* → *il compenso* (82.4%, *Z* = 4.3)], and morphological changes [e.g., *dovevi* → *devi*; *rimaneva* → *rimane*; *il nipotino* → *il nipote* (40.4%, *Z* = 2.1)] (Bonferroni, *p* ≤ 0.050).

Semantic errors, in turn, were significantly associated with nouns (34.9%, *Z* = 2.9) and adverbs (18.6%, *Z* = 2.8) (Bonferroni, *p* ≤ 0.050). Noun-errors included substitutions (63%, *Z* = 2.2) with visually similar (*è a metà dell’opera* → *è a mente dell’opera; *diede le dimissioni* → *diede le dimensioni) and/or morphologically related words (*un giocatore* → *un gioco*; *parole* → *parlare*). Adverb errors included significant rates of additions (e.g., *ma io sono* → *ma io non-sono*; *sempre chiusa* → *sempre più chiusa*) (30.4%, *Z* = 3.0).

Three distinct GMMs with MORPH, SEM, and BUFF errors as respective dependent variables, syntactic comprehension and production percent scores as fixed effects, and group and age as random effects were run. There was no predictive effect of syntactic skills (fixed effects) on any error type, but age (random effect) had a significant negative effect on SEM errors (*Coeff.* (15.572) = −0.022, *SE* = 0.008, *p* = 0.014, *CI* [−0.039, −0.005].

#### Effect of Grade on Semantic Errors

We eventually analyzed the effect of grade on semantic errors in each group by running a robust GMM with SEM-error proportions as the dependent variable, and the grade variable nested in the group variable as a fixed effect. The combination of group and grade correctly predicted semantic errors [*F*(5, 16) = 2.938, *p* = 0.045]. In third grade, DD children made significantly more semantic errors than TD children (*Est* = 0.033, *SE* = 0.013, *p* = 0.026). There was a significant decrease of semantic errors between third and fourth grade in the DD group only (*Est* = 0.075, *SE* = 0.009, *p* = 0.000) (for complete statistics, see Supplementary Tables 6–8).

## Discussion

### Differences Between Groups

We analyzed the influence of morphosyntactic skills on the reading of Italian primary-school children with DD compared to TD children. As expected, DD-children were significantly less accurate and slower in reading than TD children after controlling for decoding errors, irrespective of their age. The two groups obtained similar syntactic comprehension and production results, irrespective of their age (for qualitative response analyses of syntactic tests on a wider sample including the present participants, see Casani, 2020a,b), thus suggesting that DD does not directly affect oral morphosyntactic and syntactic skills in primary school.

The absence of effects of age on syntactic skills raised some doubts. Two additional models, where the age variable combined with the group variable was entered as a fixed effect, confirmed no effect of age on syntactic skills. The use of the grade variable instead of the age variable (combined with the group variable), on the contrary, showed significant effects on both syntactic comprehension and production. This means that children’s instructional level rather than their age affects their syntactic skills. As for syntactic comprehension, TD children revealed a significant increase in their performance until the maximum score in fourth grade. Such an increase was not present in DD children, whose score remained around 80%, and this difference between groups (in fourth grade only) was significant. This is partially consistent with studies arguing for the presence of syntactic deficits in children and adults with DD (Bishop, 1991; Muter and Snowling, 1998; Talli et al., 2013; Cardinaletti and Volpato, 2015), which might depend on the presence of undiagnosed language disorders (Guasti, 2013), or differences in DD profiles of participants. For instance, individuals with deep DD are specifically reported for possible syntactic deficits (Friedmann and Coltheart, 2018). The peculiarity of the present study is that these problems seem to be related to a specific developmental phase. Given the small number of participants distributed across grades [in fourth grade, they were 8 (5 with DD + 3 with TD)] this hypothesis should be taken cautiously and checked on larger samples. This outcome, however, stresses the importance of a careful analysis of linguistic profiles of DD children, even in a longitudinal perspective, to reveal possible problems that might emerge in particular developmental steps. A qualitative analysis of different language structures is also advisable, to reveal possible strategies that might be associated with specific developmental conditions (Casani, 2020b). In this regard, third-person direct object clitic pronouns are deemed very sensitive to detect language difficulties (e.g., Tuller et al., 2011; Varlokosta et al., 2016; Casani, 2020c), even in DD (e.g., Guasti, 2013). So, we wonder why there were no differences in their production between our groups.^3^ The answer might lie in the test used (Arosio et al., 2014), which elicits clitic pronouns under the most accessible morphosyntactic conditions, namely gender and number^4^ match between the clitic pronoun and the sentential subject (Casani and Cardinaletti, 2021). Recent studies report significant difficulties under conditions of subject-object gender mismatch (Arosio and Giustolisi, 2019; Casani and Cardinaletti, 2021). The literature describing clitic pronouns as acquired at 4 or 5 years in typically developing monolingual children (Schaeffer, 2000; Arosio et al., 2014; Belletti and Guasti, 2015; Varlokosta et al., 2016) does not consider these difficulties, so their introduction in the elicitation tasks might make some differences arise. A new test with balanced match/mismatch conditions between subject and object features is in preparation, which will help disentangle this issue (see Casani and Cardinaletti, 2021).

### Predictors of Reading

Decoding accuracy was predicted by morphosyntactic comprehension and production, with a stronger effect of comprehension, as well as by three error categories, i.e., MORPH, SEM, and BUFF errors, which have to do with morphology to different extents. MORPH errors had a very strong effect, followed by SEM and, lastly, BUFF errors.

Decoding speed, instead, was predicted by the presence of DD and by MULTI errors, namely errors in decoding multi-letter combinations, which are often non-shallow. The significant (fixed) effect of group only on decoding speed means that speed is a specific problem of DD-children and, differently from accuracy, is not directly mediated by morphosyntactic skills. This is in line with studies considering speed as a more reliable measure of DD than accuracy in shallow-orthography languages (see Zoccolotti et al., 2005). At the same time, the predictive effect of MULTI errors on decoding speed encourages the adoption of a multicomponent approach to reading, in which accuracy and speed interact. In this view, some reading errors might depend on impairments to specific sections of the reading model, which slow down the reading performance by hampering faster processing via the direct route. In this regard, a word decoding assessment battery based on the dual-route model (Friedmann and Gvion, 2003) has been recently adapted to the Italian language by Traficante et al. (2017). Their pilot study found six different types of DD in 52 Italian poor readers compared to 210 typical readers from the second to the fifth grade of primary school, thus showing that it is possible to discriminate several types of reading impairments due to selective segments of the dual-route model of reading even in a shallow-orthography language like Italian. Casani (2019) applied a coding scheme based on Friedmann and Coltheart (2018) to the text reading (Cornoldi and Colpo, 2011) of 21 children with DD, 4 of which with a developmental language disorder, compared to 32 typically developing children from the first to the second grade. That study detected 11 different error types. Children with language disorders presented the most compromised situation, with significant error proportions due to a combined deficit in the sublexical route and the orthographic visual analysis stage. The analyses of individual performances confirmed an impairment in both the lexical and sublexical route in 3 out of 4 children with language disorders. Interestingly, both Traficante et al. (2017) through the word lists, and Casani (2019) through text reading, found 11 and 2 cases of poor readers, respectively, who had not been detected through standard measures. These data encourage a fine-grained decoding error analysis even in shallow-orthography languages. At the same time, the problematic conditions of decoding skills in children with language disorders, who are likely to present with deficits in the production of direct object clitic pronouns, confirm the interrelation between reading and language skills, as well as the importance of outlining an accurate linguistic profile of subjects during DD diagnoses. The predictive role of morphosyntactic skills on reading accuracy emerged in the present study confirms this need.

The present study also revealed that the reading performance is mainly slowed down (in both DD and TD children) by MULTI errors, namely the decoding of multi-letter combinations. This supports a multicomponent approach to reading, in which decoding accuracy and speed interact with each other and with language processes. In light of a dual-route approach to orthographic processing (Grainger and Ziegler, 2011), reading accuracy is increased by familiarity with *fine-grained* representations coding for the presence of frequently co-occurring letter combinations, namely higher-level orthographic representations preserving the information about letter ordering. Morphemic constituents belong to this category. Higher morphosyntactic competence might increase children’s familiarity with these *fine-grained* representations. This might not be sufficient, however, to speed up the processes further. To this aim, children need to increase *diagnosticity*, namely to rapidly map orthography to semantics by selecting letter combinations that are most informative with respect to word identity, according to the distributional properties of word features. This is possible by processing *coarse-grained* representations, which code for the presence of informative letter combinations in the absence of precise positional information (Grainger and Ziegler, 2011; Traficante et al., 2018), as in the case of multi-letter combinations.^5^ In this interactive view of accuracy and speed, reading would be the product of orthographic, morphosyntactic, and lexico-semantic processes.

#### Morphosyntactic Predictors of Reading Accuracy

To explore in more depth the morphosyntactic processes involved in reading accuracy, we analyzed which syntactic-comprehension structures predict decoding accuracy. A sentence requiring the interpretation of a third-person direct object clitic pronoun [*La mucca le__3r*d person_fem_plur_clit*_ sta guardando* (“The cow them is watching” = The cow is watching them)], in particular, predicted decoding accuracy. Both comprehension and production of third-person direct object clitic pronouns, then, predict decoding accuracy. This might be due to the particular status of third-person direct object clitic pronouns, which match weak phonological salience to a high load of morphosyntactic information, as they are marked for person, gender, number, and case. Moreover, they are subject to syntactic movement and are placed preverbally with finite verbs, which is a non-canonical object position. Finally, they are mandatory in some contexts, forbidden in others, and optional in some others. These characteristics make third-person direct object clitic pronouns particularly sensitive to reveal language difficulties. In this regard, cross-linguistic literature reports clitic pronouns as a clinical marker of atypical language development in several languages including Italian (for Italian, see Bortolini et al., 2006; Arosio et al., 2014; for French, see Jakubowicz et al., 1998; Tuller et al., 2011; for a cross-linguistic study on 16 languages, see Varlokosta et al., 2016), as well as vulnerable in bilinguals that are scarcely exposed to the target language (for Italian, see Vender et al., 2016, 2018; Casani, 2020c; Casani and Cardinaletti, 2021). At the same time, clitics’ properties might expose them to be easily overlooked for their scarce phonological salience or avoided for their morphosyntactic difficulties during reading.

### Reading Errors

As for reading errors made by the two groups, the DD group made significantly more BUFF (phonological-output-buffer) errors than the TD group. This does not necessarily imply the presence of phonological-output-buffer dyslexia in our DD-sample but reveals a specific difficulty of DD-children in decoding longer and morphologically complex words. Other error types did not differ significantly between groups (Figure 3). VIS and NEGL errors were numerically higher in TD children, but proportions were small and the difference between groups was non-significant. Given the high standard deviation, we analyzed the association between these error types and participants. VIS and NEGL errors were significantly associated with two different children of the TD group attending the fifth grade (age = 10;4) and third grade (age = 8;10), respectively. This suggests some difficulties in the visual-orthographic analysis stage of reading, at the top of the reading model (see Figure 1), which should be carefully evaluated to exclude or confirm the possible presence of peripheral dyslexia in these two children.

#### Morphological Reading Errors

Analyses of the parts of speech affected by morphological errors revealed significant difficulties in decoding function words, i.e., determiners, clitic pronouns, and prepositions, but also verbs. The presence of verbs as the only lexical part of speech that was significantly affected by MORPH errors is consistent with studies reporting differences in lexical decision times for nouns, adjectives, and verbs (for Italian, see Colombo and Burani, 2002; Traficante and Burani, 2003; for Serbo-Croatian, see Kostić and Katz, 1987; for Hebrew, see Deutsch et al., 1998; for adults with acquired language disorders, see a review in Crepaldi et al., 2011). Deutsch et al. (1998) ascribe these processing differences, besides syntactic and semantic components, to the distributional properties of constituents, namely to the fact that “when a morpheme is common to more words in the language, its impact on processes of morphological decomposition is prominent” (Deutsch et al., 1998, p. 1,252). Traficante et al. (2018) suggest that the processing of Italian verbs might be deemed as more demanding than that of nouns because Italian verbs belong to a larger morphological family, as verb roots are shared by about 50 different inflected forms and several derived words, whereas noun roots are inflected in up to four different ways and are shared by fewer derivations. The authors refer to fMRI studies highlighting stronger activation of the left inferior frontal gyrus associated with longest reaction times during a grammatical-class switching task (for adult skilled readers, see Marangolo et al., 2006; Berlingeri et al., 2008; for subjects with Parkinson’s Disease, see Di Tella et al., 2018; Silveri et al., 2018) to conclude that processing difficulties might be due to the complexity of selection and inhibition processes required by the task. These reasons might explain the significant presence of MORPH errors in verbs in our sample.

It is worth noting that both phonologically weak (clitic pronouns, determiners, prepositions) and phonologically salient words (verbs) are significantly associated with decoding MORPH errors. This means that these errors might depend on both phonological and morphosyntactic weakness. The fact that oral comprehension and production of direct object clitic pronouns (which are phonologically weak and morphosyntactically complex) contribute significantly to reading accuracy (see above) supports this idea.

#### Semantic Errors and Interaction With Morphosyntactic Processes

SEM errors were significantly associated with nouns and adverbs. The co-occurrence of MORPH and SEM errors can be due to deep dyslexia (Friedmann and Coltheart, 2018), namely an impairment in both the lexical and sublexical route of reading. In our sample, MORPH and SEM errors correlate within the DD group (*ϱ_*s*_* = 0.564, *p* = 0.036) but not within the TD group (*p* = 0.538), thus suggesting the possible presence of deep dyslexia in the DD sample. The co-presence of comparable rates of MORPH and SEM errors in the TD group as in the DD group (see Figure 3), however, suggests that these errors might be affected by language competence. Generalized mixed analyses showed that syntactic skills do not predict any of these two error types. As for syntactic comprehension, this might be due to the nature of the structures investigated, which mainly involve general syntactic competence, except item number 5, which involves a functionally specific structure as a third-person direct object clitic pronoun. The use of a morphosyntactic-comprehension test specifically built on the structures that revealed a significant association with MORPH and SEM errors might give more informative outcomes.

Higher age predicted fewer SEM errors. This might be because children tend to read via the lexical route as their age (and expertise) increases. The core of the lexical route of reading consists of two lexicon storages containing the orthographic and the phonological lexicon, respectively. A known orthographic string activates the correspondent entry in the input orthographic lexicon. This lexicon is organized by written word frequency. Hence, compared to words with similar orthographic (and phonological) properties, the more frequent, the more accessible words are. The activated lexical representation in the input orthographic storage can be either processed through the semantic system assigning meaning to the read word or directly sent to the phonological output lexicon assigning phonological information to the read known word. The direct connection between the two lexicon storages, possibly mediated by the semantic system, allows for both accurate and faster conversion (Coltheart et al., 2001; Castles, 2006). According to the *self-teaching hypothesis* of phonological decoding (Perry et al., 2013), the activation of preexisting words in the phonological lexicon allows for the creation of orthographic entries, so that phonology works as a self-teaching device (or “built-in teacher”) refining and strengthening the network of letter-sound connections. The teaching signal that is internally generated by phonology contributes to increasing the decoding network (Ziegler et al., 2020). This might explain the predictive effect of age on the decrease of SEM decoding errors, mainly due to the “built-in taught” interactive development of phonology and lexicon thanks to the increased reading experience. We found a significantly higher rate of SEM errors by DD children in third grade, followed by a significant decrease between third and fourth grade, which equated their SEM-error rate and that of TD children. This suggests that DD might be responsible for slower development of the lexical route of reading. Given the small number of participants across grades, however, this result should be verified in larger samples, as it might be differently affected by different types of DD.

Taken together, these data highlight the interactive role of lexical skills and reading skills. In this regard, a recent longitudinal study (Casani, in preparation, 2021) confirmed predictive effects of lexical and syntactic skills on written decoding. Moreover, it found a significant role of school-grade in vocabulary acquisition but no evident effects of school-grade on more complex syntactic abilities, i.e., clitic production under the condition of increasing morphosyntactic difficulties (for information on the test used, see Casani and Cardinaletti, 2021) between the last year of kindergarten and the second grade of primary school in mono and bilingual children. A significant increase in children’s receptive vocabulary was evident only between kindergarten and second grade but not in first grade. The author suggests that the instructional input received in primary school, which also entails a certain metalinguistic component, had a role in the development of children’s vocabulary.

Given these data, the mentioned self-teaching hypothesis, and the body of research proving the effects of vocabulary on school achievements (for a review, see Elleman et al., 2009), we can motivate an interactive development of vocabulary and reading, with reciprocal effects. In this light, reading is the multicomponent product of interactive processes including phonological, morphological, morphosyntactic, and semantic skills. Similar to what happens in the interactive development of phonemic awareness and reading (see Casani, in preparation, 2021), good morphosyntactic and lexical skills might improve fast recognition of function words as well as morphological strings, such as free and bound morphemes and compound constituents; at the same time, increased abilities to recognize these strings might improve their oral mastering. In this framework, an important role in reading lies in the interaction among morpho-semantic, morpho-orthographic (Feldman et al., 2009), and morpho-syntactic processes, whose successful cooperation should facilitate morpho-lexical access.

## Conclusion

The present study demonstrates that oral morphosyntactic skills play a role in reading accuracy for both DD and TD children in a shallow-orthography language like Italian. Consistent with the dual-route approach to orthographic processing (Grainger and Ziegler, 2011), reading accuracy was mediated by morphosyntactic competence, which helped process *fine-grained* orthographic representations maximizing precise ordering of letters, such as morphemic constituents. On the other hand, reading speed was mainly affected by familiarity with *coarse-grained* orthographic representations coding for the presence of informative letter combinations without precise positional information (Grainger and Ziegler, 2011; Traficante et al., 2018), such as multi-letter combinations.

Third-person direct object clitic pronouns were confirmed as a sensitive structure not only for oral language but also for written language, as both their comprehension and production predicted decoding accuracy. We attributed this sensitivity to the fact that clitics match weak phonological salience with a heavy load of morphosyntactic information. Direct object clitic pronouns, determiners, and prepositions, as well as verbs, were significantly associated with MORPH decoding errors, thus suggesting that these errors are due to both phonological and morphosyntactic competence.

Age predicted a decrease of semantic decoding errors, meaning that children generally tend to read via the lexical route as their age increases. This is consistent with the development of the orthographic step of reading (Frith, 1985), namely of *fine-grained* chunks (Grainger and Ziegler, 2011) to be processed via the lexical route (Coltheart et al., 2001). We hypothesized that DD might slow down the acquisition of these processes as well as morphosyntactic skills. In any case, chunking the text into meaningful orthographic strings, which include free and bound morphemes, function words, morphological-compound constituents, as well as frequent multi-letter combinations, improves the reading performance by reducing the number of units to be processed (Traficante et al., 2018). Increased familiarity with these units and their distribution, deriving from higher morphosyntactic, lexical, and orthographic competence might improve their decoding.

Since children tend to read via the lexical route as their expertise increases, interventions to enhance their familiarity with functional strings should be planned timely to avoid resorting to inadequate lexical-orthographic compensation strategies, which are required by increasingly demanding texts proposed in school. This might facilitate, in particular, the reading of longer and morphologically complex words, in which DD children revealed particular difficulties, through the decomposition into phonologically and semantically meaningful chunks to be processed via the lexical route.

These data argue in favor of a multicomponent approach to reading, in which linguistic, metalinguistic, orthographic, and cognitive skills interact. In the Italian shallow orthography, morphological competence can affect decoding since the second grade (for studies on the role of morphological awareness in Italian reading, see Burani et al., 2002, 2008; Marcolini et al., 2011; Traficante et al., 2011; for a longitudinal study showing the role of (morpho)syntactic competence in the reading of Italian mono and bilingual children, see Casani, in preparation, 2021). It is then advisable to assess the linguistic profile of children during DD diagnoses to establish whether some reading errors are related to morphosyntactic difficulties. In these cases, in particular, a morphosyntactic training aiming at recognizing function elements, which might be easily mistaken for their morphological complexity and/or overlooked for their phonological weakness, might be useful to increase reading accuracy. Longitudinal intervention studies might support this statement. At the same time, cross-sectional studies are needed to explore the age of impact of morphology on reading in languages with different morphological richness and orthographic depth.

## Data Availability Statement

The datasets presented in this article are not readily available because they include participants’ sensitive data. Requests to access the datasets should be directed to EC, emanuele.casani@unive.it.

## Ethics Statement

The studies involving human participants were reviewed and approved by Ca’ Foscari University of Venice. Written informed consent to participate in this study was provided by the participants’ legal guardian/next of kin.

## Author Contributions

EC collected and analyzed the data, and prepared the first draft and revisions. MV and AC participated in data discussion and edited the manuscript. All authors listed have made a substantial, direct, and intellectual contribution to the work, and approved it for publication.

## Conflict of Interest

The authors declare that the research was conducted in the absence of any commercial or financial relationships that could be construed as a potential conflict of interest.

## Publisher’s Note

All claims expressed in this article are solely those of the authors and do not necessarily represent those of their affiliated organizations, or those of the publisher, the editors and the reviewers. Any product that may be evaluated in this article, or claim that may be made by its manufacturer, is not guaranteed or endorsed by the publisher.

## Footnotes

1 The syntactic-comprehension test (Bisiacchi et al., 2005) includes separate standard scores for grammar-focused items and lexicon-focused items, thus allowing to evaluate the two areas independently, at least in the authors’ intentions. In our opinion, however, the test should be restandardized by considering item number 5 among grammar-focused items (see the section “Analyses”).

2 We have not commented on the significant increase of morphosyntactic-comprehension scores found in the DD group between second and third grade (see Supplementary Table 2) because it is based on one second-grader only and is not comparable with the TD group, where there were no second-graders (see the number of participants in each grade in Supplementary Table 1).

3 There was only an intra-group difference revealing a significant increase of target clitics between fourth and fifth grade in the DD group, which might suggest a different acquisitional pace between groups.

4 Subject-object number mismatch showed not to be as problematic as gender mismatch for third-person direct object clitic production (Casani and Cardinaletti, 2021).

5 Frequent multi-letter compounds can enter the orthographic lexicon and be processed via the *fine-grained* route (Grainger and Ziegler, 2011) in skilled readers. In any case, their processing would not be mediated by morphosyntactic competence as the processing of morphological constituents.
