# Supplementary material for: Morphosyntactic Skills Influence the Written Decoding Accuracy of Italian Children With and Without Developmental Dyslexia
Source: Front Psychol. 2022 Apr 27;13:841638. doi: 10.3389/fpsyg.2022.841638 (PMC9094683; doi:10.3389/fpsyg.2022.841638)
Supplement: Supplementary file 1 [file Data_Sheet_1.pdf]

Supplementary Material

1 Supplementary Data

The following grammar-focused items of the syntactic-comprehension test (Bisiacchi et al., 2005) were analyzed:

(item 5) a 3rd-person direct object clitic pronoun [*La mucca* ***le*** *sta guardando* (The cow **them** is watching = The cow is watching them)];

(item 9) a lexical majority comparative [*il coltello è più lungo della matita* (the knife is more long than the pencil → the knife is longer than the pencil)];

(item 10) a passive voice [*L'elefante è spinto dal ragazzo* (The elephant is pushed by the boy)];

(item 11) an articulated spatial preposition [*La matita è sulla*_$_{\text{fem.sing.art.prep.}}$ *scatola* (The pencil is onthe box → The pencil is on the box)];

(item 12) a center-embedded subject relative clause [*Il ragazzo che sta inseguendo il cavallo è grasso* (The boy who is chasing the horse is fat)];

(item 13) an incidental adversative negative clause [*Il cavallo, ma non il ragazzo, sta in piedi* (The horse, but not the boy, is standing)].

(item 14) a spatial preposition [*La matita è sopra il fiore* (The pencil is above the flower)];

(item 15) an adversative correlative sentence [*Non solo la ragazza ma anche il gatto è seduto* (Not only the girl but also the cat is sitting)];

(item 16) a right-branching subject relative clause [*La ragazza insegue il cane che è grande* (The girl chases the dog which is big → The girl is chasing the dog which is big)];

(item 17) a negative correlative sentence [*Né il ragazzo né il cavallo stanno correndo* (Neither the boy nor the horse are running);

(item 18) a reduced passive relative clause [Il ragazzo inseguito dal cane è grande (The boy chased by the dog is big)].

The items are reported as they are written in the administration protocol. They were read by the administrator without prosodic cues. Item 13, in particular, was read without perceptible commas. Children were required to choose the matching picture among four possible solutions. Pictures were printed in grey scales.

2 Supplementary Tables

Supplementary Table 1. Syntactic-comprehension estimates for each group in each grade.

					CI (95%)	
Group	Grade	Subjects N	*Mean*	*SE*	Lower	Upper
CG	3	14	0.788	0.035	0.720	0.862
	4	3	0.970	0.024	0.921	1.021
	5	11	0.901	0.027	0.848	0.957
DD	2	1	0.640	0.000	0.640	0.640
	3	2	0.865	0.032	0.803	0.932
	4	5	0.802	0.064	0.681	0.944
	5	6	0.874	0.032	0.811	0.942

Supplementary Table 2. Intra-group significant increase in syntactic-comprehension scores.

							CI (95%)	
Group	Grade contrast	*Est.*	*SE*	*t*	*Df**	*p***	Lower	Upper
CG	3 - 4	- 0.182	0.043	- 4.284	33	0.000	- 0.269	- 0.096
DD	2 - 3	- 0.225	0.032	- 7.071	33	0.000	- 0.290	- 0.160

* Satterthwaite appr.
** $p = 0.05$ adapted to the least significant difference

Supplementary Table 3. Significant difference between groups in syntactic comprehension.

							CI (95%)	
Group contrast	Grade	*Est.*	*SE*	*t*	*Df**	*p***	Lower	Upper
CG - DD	4	0.168	0.069	2.438	33	0.020	0.028	0.308

* Satterthwaite appr.
** $p = 0.05$ adapted to the least significant difference

Supplementary Table 4. Clitic-production estimates for each group in each grade.

					CI (95%)	
Group	Grade	Subjects N	*Mean*	*SE*	Lower	Upper
CG	3	14	0.959	0.024	0.911	1.010
	4	3	0.887	0.046	0.797	0.986
	5	11	0.932	0.026	0.880	0.986
DD	2	1	0.830	0.000	0.830	0.830
	3	2	0.835	0.060	0.721	0.967
	4	5	0.818	0.059	0.706	0.948
	5	6	0.972	0.026	0.920	1.026

Supplementary Table 5. Intra-group significant increase in clitic-production scores.

							CI (95%)	
Group	Grade contrast	*Est.*	*SE*	*t*	*Df**	*p***	Lower	Upper
DD	4 - 5	- 0.154	0.065	- 2.380	31	0.024	- 0.285	- 0.022

* Satterthwaite appr.
** $p = 0.05$ adapted to the least significant difference

Supplementary Table 6. Estimates of reading semantic errors for each group in each grade.

					CI (95%)	
Group	Grade	Subjects N	*Mean*	*SE*	Lower	Upper
CG	3	14	0.100	0.013	0.075	0.133
	4	3	0.085	0.034	0.036	0.200
	5	11	0.060	0.016	0.034	0.106
DD	2	1	0.067	0.000	0.067	0.067
	3	2	0.133	0.000	0.133	0.133
	4	5	0.058	0.009	0.042	0.081
	5	6	0.125	0.037	0.067	0.233

Supplementary Table 7. Intra-group significant decrease in reading semantic errors.

							CI (95%)	
Group	Grade contrast	*Est.*	*SE*	*t*	*D**	*p***	Lower	Upper
DD	3 - 4	0.075	0.009	8.305	16	0.000	0.056	0.094

* Satterthwaite appr.
** *p* = 0.05 adapted to the least significant difference

Supplementary Table 8. Significant difference between groups in reading semantic errors.

							CI (95%)	
Group contrast	Grade	*Est.*	*SE*	*t*	*Df**	*p***	Lower	Upper
CG - DD	3	- 0.033	0.013	- 2.449	16	0.026	- 0.062	- 0.004

* Satterthwaite appr.
** *p* = 0.05 adapted to the least significant difference
